# Torsion of Undescended Third Testis, as Rare Cause of Painful Inguinal Mass

**DOI:** 10.1155/2015/273508

**Published:** 2015-01-26

**Authors:** Suheil Artul, Faozi Artoul, Basel Fahoum, William Nseir, Najib Nasrallah, George Habib

**Affiliations:** ^1^Department of Radiology, EMMS Hospital, 16100 Nazareth, Israel; ^2^Faculty of Medicine in the Glilee, Bar-Ilan University, 13115 Safed, Israel; ^3^Department of Nuclear Medicine, Meir Hospital, 44410 Betah Tekva, Israel; ^4^Urology Department, EMMS Hospital, 16100 Nazareth, Israel; ^5^Medical Department, Carmel Medical Center, Haifa, Israel

## Abstract

Twenty years old young was referred to our department due to painful inguinal mass. The mass was diagnosed as torsion of third testis which was treated by orchiectomy. Polyorchidism is a rare entity with increased risk for malignancy and torsion.

## 1. Introduction

Polyorchidism is a rare condition; it happens due to an embryological abnormal division of the genital ridge [1].

These supernumerary testes are at increased risk to develop malignancy and torsion [1, 2].

Triorchidism is the most common form, but also 4/5 testes have been reported in literature.

The supernumerary testes are usually intrascrotal [1].

The minority of all reported cases are inguinal and some of them even retroperitoneal.

The third testis is usually more mobile and more prone to torsion [3].

Ultrasound colour plays a crucial role in diagnosing and following up this entity. MRI plays a role when the diagnosis is in doubt or when suspecting an associated malignancy.

Some patients after bilateral orchiectomy remained reproductive because of missing the undiagnosed third undecsended testis. In fact this happens because some of the supernumerary testis has attachment to a draining epididymis and vas deferens [1–3].

## 2. Case Report

Twenty years old young, was referred to our ultrasound unit because of a history of two-day painful right inguinal mass. The patient which is usually healthy had no fever in these two days.

Physical examination revealed a tender mass in the right inguinal area, the patient had no fever, and laboratory tests were in normal range.

**Figure 1:**
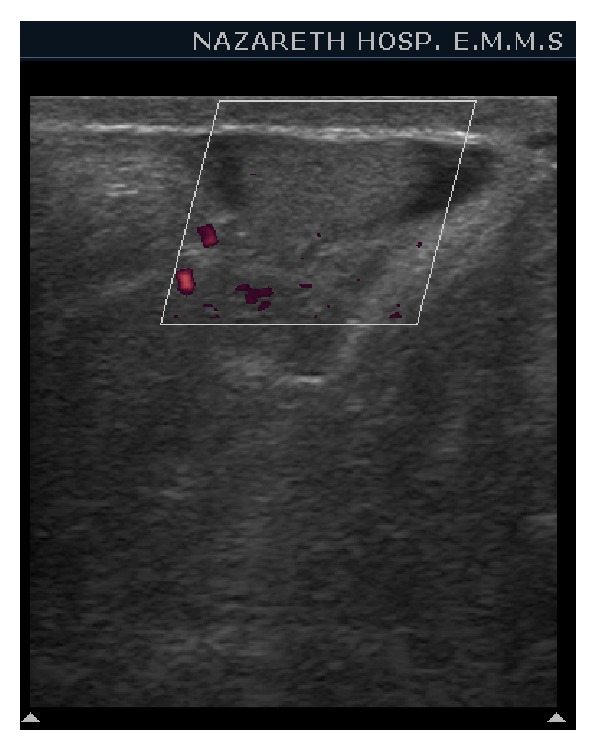
Ultrasound color Doppler of the inguinal area shows an inguinal oval 1.6 cm, hypoechogenic mass with no flow in it that resembles small testis.

**Figure 2:**
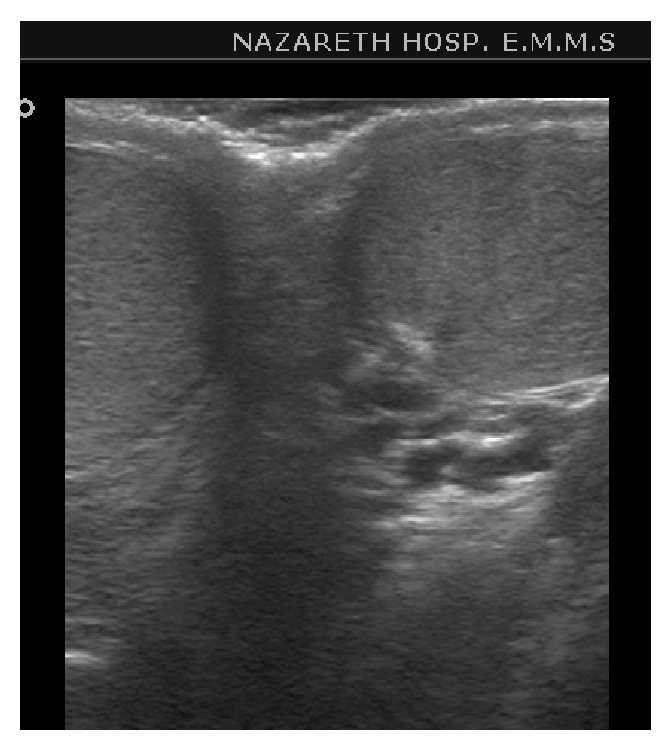
Transverse ultrasound of scrotum shows normal two testes in place.

Ultrasound of the inguinal area showed an inguinal oval 1.6 cm, echogenic mass with no flow in it that resembles small testis (Figure 1). Ultrasound of the scrotum showed normal two testes in place (Figure 2). Therefore the diagnosis of polyorchidism was done with torsion of the third undescended testis. The diagnosis was confirmed at surgery and resection of the ischemic third inguinal testicle was done.

## 3. Discussion

Embryologically polyorchidism can be classified into four types [4]. In type A, the division separates a small part of the genital ridge, which does not contact the mesonephric duct. Therefore, the supernumerary testis lacks an epididymis and vas deferens; this type is more prone to movement and can be presented as undescended testis such as our case. In type B, the division of the genital ridge occurs in the region where the primordial gonads are attached to the mesonephric ducts and the supernumerary testis has its own epididymis. In type C, the supernumerary testis has its own epididymis and shares the vas deferens with the regular testis in a parallel fashion. In this type of polyorchidism, there is an incomplete longitudinal division of the genital ridge and the proximal portion of the mesonephric duct. In type D, which is the least common, complete longitudinal duplication of the genital ridge and mesonephric duct occurs, with resultant complete duplication of testes, epididymides, and vas deferens.

Triorchidism is the most common type of polyorchidism and presents with two testes on one side (usually the left) and one testis on the other side. Rare case of polyorchidism with three homolateral testes on the right side and absent testis on the left side has been reported [1, 4].

Splenogonadal fusion is a rare congenital anomaly that may sonographically resemble polyorchidism [5]. In this entity spleen, gonad, epididymis, and vas deferens are fused. Sonography reveals a mass with the testicle of similar echogenicity and it may mimic the appearance of polyorchidism. When splenogonadal fusion is suspected, a technetium sulfur colloid scan should be performed to confirm the presence of ectopic splenic tissue. Another classification is based on reproductive potential of the supernumerary testis. In type 1, the supernumerary testis has reproductive potential because of attachment to a draining epididymis and vas deferens. In type 2, the supernumerary testis has no reproductive potential because of lack of a draining system. The sonographic appearance of polyorchidism is presence of scrotal mass that has an echo pattern identical to that of the ipsilateral testicle [6] MRI appearance is a round or oval shaped structure showing typical signal characteristics of testicles, that is, homogeneous intermediate signal intensity on T1 weighted and high signal intensity on T2 weighted images [7].

Polyorchidism is a rare genitourinary abnormality, and torsion is one of its associated complications; however, the diagnosis of polyorchidism with or without torsion can be made readily with ultrasonography when one is aware of this entity [8].

Sonographic features of torsion are homogenous hypoechogenicity of testis with absent flow on color Doppler study. MRI can play a role in diagnosing torsion; signs in MRI are increased signal intensity on T1 and decreased signal intensity on T2 weighted sequences [9].

## 4. Conclusion

- Not every inguinal mass is a hernia or a lymph node.
- Polyorchidism is a rare congenital anomaly.
- Torsion is a complication of this entity.
- Ultrasound Doppler plays a crucial rule in diagnosing.
- If you do not think about it, you will not mention it in your diagnosis.
